# TGF-β-Elicited Induction of Tissue Inhibitor of Metalloproteinases (TIMP)-3 Expression in Fibroblasts Involves Complex Interplay between Smad3, p38α, and ERK1/2

**DOI:** 10.1371/journal.pone.0057474

**Published:** 2013-02-28

**Authors:** Suvi-Katri Leivonen, Konstantinos Lazaridis, Julie Decock, Andrew Chantry, Dylan R. Edwards, Veli-Matti Kähäri

**Affiliations:** 1 Department of Dermatology, University of Turku, and Turku University Hospital, Turku, Finland; 2 MediCity Research Laboratory, University of Turku, Turku, Finland; 3 School of Biological Sciences, University of East Anglia, Norwich, United Kingdom; University of Patras, Greece

## Abstract

Transforming growth factor-β (TGF-β) promotes extracellular matrix deposition by down-regulating the expression of matrix degrading proteinases and upregulating their inhibitors. Tissue inhibitor of metalloproteinases (TIMP)-3 is an ECM-associated specific inhibitor of matrix degrading metalloproteinases. Here, we have characterized the signaling pathways mediating TGF-β-induced expression of TIMP-3. Basal and TGF-β-induced TIMP-3 mRNA expression was abolished in Smad4-deficient mouse embryonic fibroblasts and restoring Smad4 expression rescued the response. Inhibition of Smad signaling by expression of Smad7 and dominant negative Smad3 completely abolished TGF-β-elicited expression of TIMP-3 in human fibroblasts, whereas overexpression of Smad3 enhanced it. Inhibition of extracellular signal-regulated kinase 1/2 (ERK1/2) activation with PD98059 and p38 mitogen-activated protein kinase activity by SB203580 resulted in suppression of TGF-β-induced TIMP-3 expression, indicating that ERK1/2 and p38 MAPK mediate the effect of TGF-β on TIMP-3 expression. Specific activation of p38α and ERK1/2 by constitutively active mutants of MKK3b or MEK1, respectively, and simultaneous co-expression of Smad3 resulted in induction of TIMP-3 expression in the absence of TGF-β indicating that Smad3 co-operates with p38 and ERK1/2 in the induction of TIMP-3 expression. These results demonstrate the complex interplay between Smad3, p38α, and ERK1/2 signaling in the regulation of TIMP-3 gene expression in fibroblasts, which may play a role in inflammation, tissue repair, and fibrosis.

## Introduction

Proteolytic turnover of extracellular matrix (ECM) is an essential feature of connective tissue remodeling during embryonic development, angiogenesis, and tissue repair. On the other hand, excessive breakdown of ECM, due to an imbalance between the activity of matrix degrading proteinases and their inhibitors apparently play an important role in many pathological conditions, such as arthritis, fibrosis and cancer invasion and metastasis [1], [2]. Matrix metalloproteinases (MMPs) are a family of zinc-dependent metalloendopeptidases collectively capable of degrading essentially all ECM components [3]. Tissue inhibitors of metalloproteinases (TIMPs) are specific endogenous inhibitors of MMP activity. They bind MMPs non-covalently in 1∶1 stoichiometric complexes and interact directly with the active sites of MMPs. The vertebrate TIMP family consists of four members: TIMP-1, TIMP-2, TIMP-3, and TIMP-4 [4]. TIMP-3 is retained in the ECM, whereas other TIMPs are secreted in soluble form. TIMPs inhibit the activity of all MMPs, although there are differences in their inhibitory profiles. TIMP-1 inhibits the activity of most MMPs, with the exception of MT-MMPs and MMP-19 [5]. In addition, TIMP-1 inhibits ADAM-10 (proteinase with A Disintegrin And Metalloprotease domain). TIMP-2, TIMP-3, and TIMP-4 inhibit all MMPs, but with different binding affinities. TIMP-3 also inhibits the activity of ADAM-17 (tumor necrosis factor-α (TNF-α) converting enzyme (TACE)), ADAM-12, ADAM-TS4 (aggrecanase-1) and ADAM-TS5 (aggrecanase-2) [5]. Furthermore, TIMPs form complexes with proMMPs and regulate their activation. TIMP-3 has been shown to promote apoptosis in several types of normal and malignant human cells in culture and *in vivo* [6]–[10], and thereby suppresses tumor growth. TIMP-3 gene expression in cultured cells is induced by mitogenic stimuli, *e.g*., serum, epidermal growth factor (EGF), and transforming growth factor-β (TGF-β), [11]–[14]. In addition, TIMP-3 expression is induced in fibroblasts in scleroderma skin, suggesting a role for TIMP-3 in dermal fibrosis [15].

TGF-β is a multifunctional growth factor controlling cell growth and differentiation, and it has marked effects on ECM deposition [16], [17]. TGF-β induces ECM gene expression and suppresses the expression of many matrix degrading proteinases, including MMP-1 in fibroblasts [18], [19]. The cellular effects of TGF-β are mediated via Smad and mitogen-activated protein kinase (MAPK) signaling pathways [20]. TGF-β-activated Smads are subgrouped into three groups according to their function: receptor-activated Smads (Smad2 and Smad3), common-mediator Smad (Smad4), and inhibitory Smad (Smad7). Receptor-activated Smad2 and Smad3 are phosphorylated by the activated TGF-β receptor complex. Following phosphorylation these Smads associate with Smad4, and are translocated to the nucleus, where Smads bind to DNA or associate with other transcriptional co-activators or co-repressors, and regulate the transcription of TGF-β responsive genes. Smad7 is an inhibitory Smad, the expression of which is induced by TGF-β and it inhibits phosphorylation of Smad2 and Smad3 by competetively interacting with the TGF-β receptor complex.

TGF-β also activates MAPKs extracellular signal-regulated kinase (ERK1/2), c-Jun N-terminal kinase (JNK), and p38 in various types of cells [20], [21]. It has become evident that there is crosstalk between the distinct cell signaling cascades activated by TGF-β. For example, ERK1/2, JNK, and p38 MAPKs can influence the activation of the Smad pathway by phosphorylating Smad2 or Smad3 [22]–[26]. In addition, delayed phosphorylation of p38 MAPK by TGF-β is mediated by the Smad pathway via GADD45β [27]–[29].

In this study, we have characterized the cellular signaling pathways involved in regulating TIMP-3 gene expression in fibroblasts. Our results show, that TGF-β -elicited induction of TIMP-3 expression is dependent on Smad3, p38, and ERK1/2 signaling, and that these signaling pathways cooperate in the regulation of TIMP-3 expression, which may play a role in inflammation, tissue repair, and fibrosis.

## Materials and Methods

### Cell Cultures and Reagents

Normal human gingival fibroblasts were kindly provided by Dr. Lari Häkkinen (University of British Columbia, Vancouver, BC) [21], [25]. The generation of Smad4 deficient EF7KO mouse embryonic fibroblasts (MEFs) has been described before [30]. Corresponding wild-type MEFs (EF7WT) were used as control cells. The cells were grown in Dulbecco’s Modified Eagle’s Medium (DMEM; Sigma, St. Louis, MO) supplemented with 10% fetal calf serum (FCS), 2 mM L-glutamine, 100 IU/ml penicillin-G, and 100 µg/ml streptomycin. Human recombinant TGF-β1 was obtained from Sigma (St. Louis, MO), and p38 MAPK inhibitor SB203580 and MEK1/2 inhibitor PD98059 from Calbiochem (San Diego, CA).

### Transduction of Cells with Recombinant Adenoviruses

The construction of empty control virus RAdpCA3 and recombinant adenoviruses RAdSmad2, RAdSmad3, RAdSmad4 for HA-tagged Smad2, Smad3, and Smad4, respectively, has been described previously [25]. Recombinant adenoviruses for Smad7 (RAdSmad7) [31] and dominant negative Smad3 (RAdSmad3DN) [32] were kindly provided by Dr. Aristidis Moustakas (Ludwig Institute for Cancer Research, Uppsala, Sweden). Adenovirus for constitutively active MKK3b (RAdMKK3bE) and for wild type p38α (RAdp38α) [33] were kindly provided by Dr. Jiahuai Han (Scripps Research Institute, La Jolla, CA), adenovirus for constitutively active MEK1 (RAdMEK1CA) [34] by Dr. Marco Foschi (University of Florence, Italy), and control adenoviruses RAd66 and RAdLacZ [35] by Dr. Gavin W.G. Wilkinson (University of Cardiff, UK).

Adenoviral infections of human gingival fibroblasts were performed as previously described [25]. In experiments, gingival fibroblasts, EF7WT, and EF7KO cells were transduced in suspension with MOIs 500, 100, and 300, respectively. Thereafter, the cells were plated and incubated for 18 h in DMEM with 1% FCS. The medium was replaced with DMEM without FCS, and the incubations were continued for 24 h. The cultures were treated with TGF-β1 (5 ng/ml) for indicated periods of time. Thereafter, cell layers were harvested either for RNA extraction to detect TIMP-3, TIMP-1, and PAI-1 (plasminogen-activator inhibitor-1) mRNAs by quantitative reverse-transcription-PCR (qRT-PRC) analysis or Northern blotting, or for the determination of TIMP-3 from the cell lysates by Western blotting.

### Northern Blot Hybridizations

Total cellular RNA was extracted with Qiagen’s Rapid RNA Purification Kit (Qiagen, Chatsworth, CA), and Northern blot hybridizations were performed as described previously [25]. For hybridizations, a-0.6 kb cDNA of human TIMP-3 and a 0.6-kb cDNA of human TIMP-1 obtained by RT-PCR as previously described [15], were used. Human plasminogen activator inhibitor (PAI-1) cDNA [36], and a 1.3-kb rat GAPDH (glyceraldehyde 3-phosphate dehydrogenase) cDNA [37] were used for detecting PAI-1 and GAPDH mRNAs, respectively.

### Quantitative Reverse Transcription PCR Analysis

Total RNAs were isolated by using the Qiagen RNeasy kit (Qiagen, Chatsworth, CA). The levels of TIMP-3 and PAI-1 mRNAs were determined by quantitative reverse transcription PCR (qRT-PCR, TaqMan®) (Table S1). Aliquots of total RNA (1 µg) were first reverse transcribed into cDNA. Taqman analysis was performed using the Applied Biosystems ABI prism 7700 sequence-detection system as previously described [38]. 18S ribosomal RNA was used as an endogenous control to normalize for differences in the amount of total RNA in each sample.

### Western Blot Analysis

Aliquots of conditioned media or cell lysates were fractionated on SDS-polyacrylamide gels, and transferred to Hybond ECL nitrocellulose membrane (Amersham Pharmacia Biotech, UK). The membranes were blocked against non-specific binding using 5% skim milk. Proteins were detected using specific primary antibodies and peroxidase conjugated secondary antibodies. Monoclonal antibody for TIMP-3 was purchased from Chemicon International Inc. (Temecula, CA) and monoclonal antibody for β-actin from Sigma (St. Louis, MO). Polyclonal antibody for Smad4 was from Santa Cruz Biotechnology (Santa Cruz, CA) and rat monoclonal anti-HA 3F10 antibody from Roche (Mannheim, Germany). The blots were visualized by enhanced chemiluminescence (ECL) detection system (Amersham Pharmacia Biotech, UK).

## Results

### TGF-β-elicited Induction of TIMP-3 Expression is Smad-dependent

**Figure 1:**
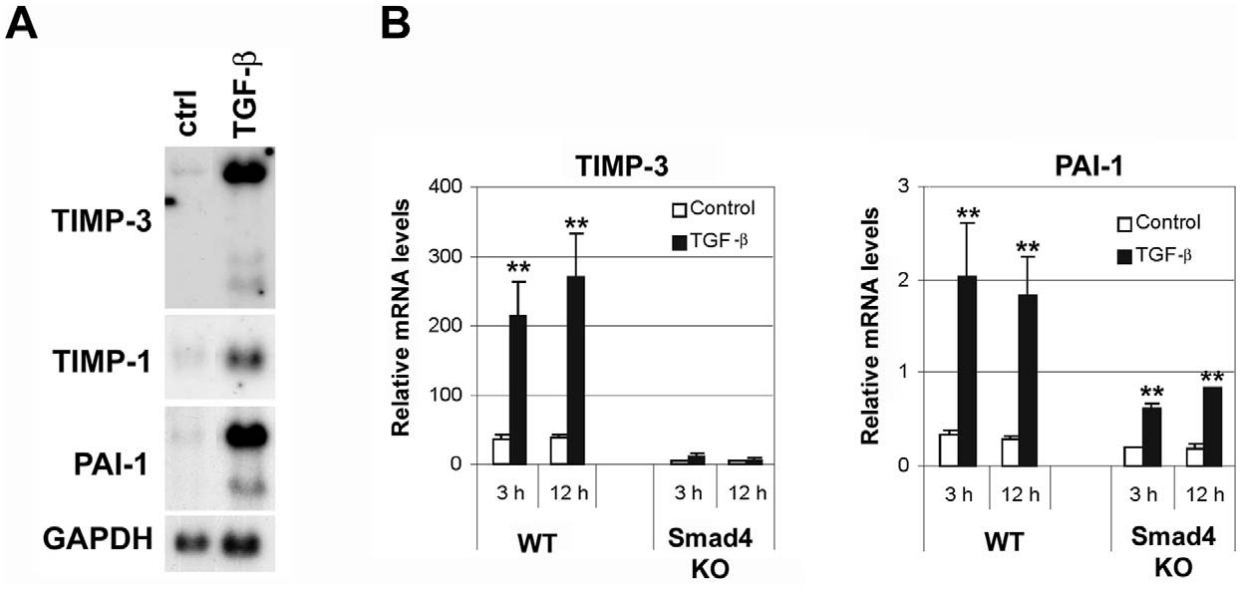
TGF-β induces TIMP-3 gene expression in a Smad-dependent manner in fibroblasts. (**A**) Human gingival fibroblasts were treated with TGF-β1 (5 ng/ml) for 24 h. Thereafter, total cellular RNAs were harvested and analyzed for the expression of TIMP-3, TIMP-1, PAI-1, and GAPDH mRNAs by Northern blotting. (**B**) EF7WT and EF7Smad4KO (Smad4 deficient) cells were treated with TGF-β1 (5 ng/ml) for 3 h and 12 h or left untreated (control). Total RNA was extracted and TIMP-3 and PAI-1 gene expression was determined by qRT-PCR. mRNA expression (mean+SD) is shown relative to 18S ribosomal RNA (n = 4). *p<0.05, **p<0.005 (t-test) for TGF-β vs. control cultures.

TGF-β has been previously shown to induce TIMP-1 and TIMP-3 gene expression in fibroblasts [12]. To confirm that TIMP-3 is a TGF-β responsive gene in human gingival fibroblasts, the cells were first treated with TGF-β1 for 24 h and analyzed for the expression of TIMP-3 mRNAs by Northern blot analysis. The TGF-β1 concentration used was 5 ng/ml, which has been shown to give maximal stimulation in fibroblasts [21], [24]–[26], [39], [40]. In addition, previous studies have shown that the response of fibroblasts to TGF-β1 in this concentration is comparable to TGF-β2 and TGF-β3 [40]–[42]. As shown in Figure 1A, TGF-β stimulation resulted in marked induction of TIMP-3, TIMP-1, and PAI-1 mRNA expression, as compared to untreated control cells. To study the Smad-dependence of the TGF-β-induced expression of TIMP-3, Smad4-deficient mouse embryonic fibroblasts, (EF7KO), and the corresponding wild type MEFs (EF7WT), were treated with TGF-β for 3 h and 12 h, as indicated. Thereafter, the expression of TIMP-3 and PAI-1 mRNAs were analyzed by qRT-PCR. As shown in Figure 1B, TGF-β stimulation of EF7WT fibroblasts resulted in potent induction of TIMP-3 and PAI-1 mRNA expression. However, in EF7KO cells the basal and TGF-β-induced expression of TIMP-3 mRNA was abolished, indicating that the Smad signaling pathway is essential for the expression of TIMP-3. In addition, the TGF-β-induced expression of PAI-1 mRNA was reduced, as compared to EF7WT control cells (Figure 1B). This is in accordance with previous observations showing that PAI-1 is a Smad-responsive gene [43], [44]. However, the TGF-β-induced PAI-1 mRNA expression was not completely abolished in EF7KO cells, indicating that other signaling pathways also participate in TGF-β induction of PAI-1 expression.

### Restoration of Smad4 expression Rescues the Induction of TIMP-3 and PAI-1 Expression in Response to TGF-β

**Figure 2:**
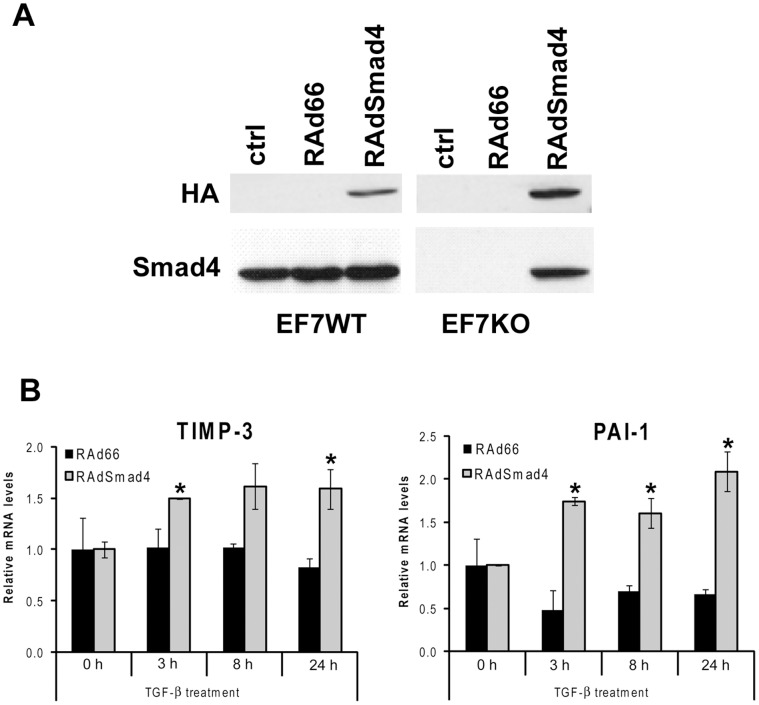
Expression of Smad4 rescues the TGF-β response of TIMP-3 and PAI-1 in Smad4 null fibroblasts. (**A**) EF7WT (wild-type) and EF7KO (Smad4 deficient) fibroblasts were transduced with recombinant adenovirus for HA-tagged Smad4 (RAdSmad4), or with empty control virus RAd66 at MOI 100 (EF7WT) or 300 (EF7KO). After 36 h incubation cell lysates were harvested and analyzed by Western blotting to detect the levels of endogenous and exogenous Smad4. Anti-HA antibody was used to detect adenovirally delivered Smad4 (upper panel) and anti-Smad4 to detect endogenous Smad4 (lower panel). (**B**) EF7KO fibroblasts were infected with adenoviruses RAdSmad4 or RAd66. After 36 h incubation the cells were stimulated with TGF-β1 (5 ng/ml) for different periods of time, as indicated. Total RNA was extracted and analyzed by qRT-PCR to determine TIMP-3 and PAI-1 mRNA levels. mRNA expression (mean ± SEM from two separate experiments, both run with duplicates) is shown relative to 18S ribosomal RNA. *p<0.05, t-test).

To further elucidate the role of Smad signaling in TGF-β-induced TIMP-3 gene expression, we analyzed whether exogenous expression of Smad4 could rescue the TGF-β response of TIMP-3 expression in Smad4-deficient MEFs. We utilized adenoviral gene delivery of recombinant adenovirus expressing HA-tagged Smad4 (RAdSmad4). EF7WT and EF7KO cells were infected with an empty control virus RAd66 or RAdSmad4 and incubated for 36 h. Thereafter, the cells were treated with TGF-β1 for different periods of time up to 24 h, and the RNAs were subjected to qRT-PCR analysis. As shown in Figure 2A, uninfected or RAd66 infected EF7KO cells expressed no Smad4, as compared to EF7WT control cells. However, Smad4 expression was restored in RAdSmad4 infected EF7KO cells, as detected with Smad4 antibody or with HA-antibody, which detects only adenovirus-produced HA-Smad4 (Figure 2A). TGF-β was unable to induce TIMP-3 or PAI-1 expression in EF7KO cells transduced with control virus RAd66, whereas restoring Smad4 expression resulted in marked and significant upregulation of TIMP-3 expression in response to TGF-β (Figure 2B). Together, these results support the observation that TGF-β-induced TIMP-3 and PAI-1 gene expression is Smad-dependent.

### TGF-β-elicited Induction of TIMP-3 Expression is Mediated by Smad3

**Figure 3:**
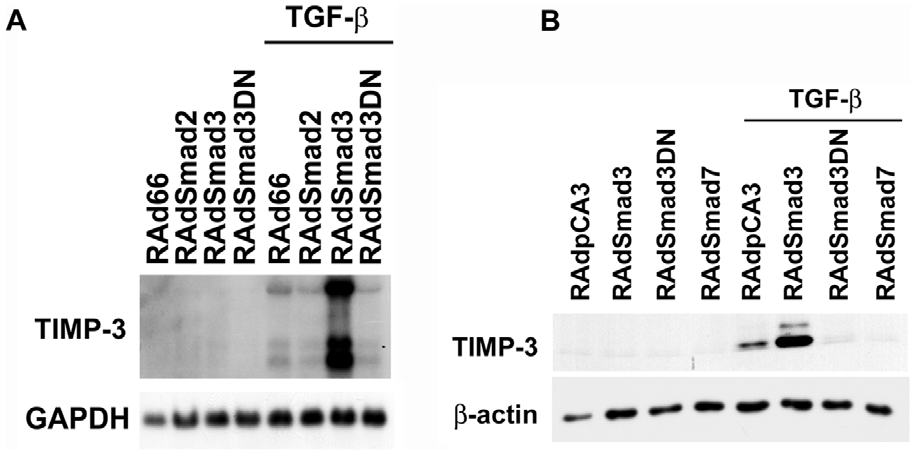
Smad3 mediates TGF-β-elicited induction of TIMP-3 expression in human fibroblasts. (**A**) Normal human gingival fibroblasts were transduced with recombinant adenoviruses for Smad2 (RAdSmad2), Smad3 (RAdSmad3), dominant negative Smad3 (RAdSmad3DN), or with empty control virus (RAd66) at MOI 500, and incubated for 18 h. Thereafter, the cells were treated with TGF-β1 for 24 h. The cell layers were harvested for RNA extraction and analyzed for the expression of TIMP-3 or GAPDH by Northern blot hybridizations. (**B**) Normal human gingival fibroblasts were infected with RAdSmad3, RAdSmad3DN, adenovirus for Smad7 (RAdSmad7), or with empty control virus (RAdpCA3) as in (**A**). Cells were treated with TGF-β1 for 24 h, the cell layers harvested and analyzed for the expression of TIMP-3 by Western blotting. Equal loading was confirmed by stripping and reprobing the same filter for β-actin.

As the results above with Smad4 deficient fibroblasts demonstrate that the TGF-β-induced expression of TIMP-3 is Smad-dependent in mouse fibroblasts, we studied this in detail also in human fibroblasts. Human gingival fibroblasts were transduced with recombinant adenoviruses for Smad2, Smad3, dominant negative Smad3 (RAdSmad2, RAdSmad3, and RAdSmad3DN, respectively), and with empty control adenovirus RAd66, and incubated for 18 h. Thereafter, the cells were treated with TGF-β for 24 h, as indicated. As shown in Figure 3A, a 24 h TGF-β stimulation of control virus RAd66 transduced cells resulted in the induction of TIMP-3 mRNA expression, as compared to untreated control cells. Interestingly, adenoviral overexpression of Smad3 markedly enhanced the TGF-β-elicited expression of TIMP-3 mRNAs (Figure 3A), whereas overexpression of Smad2 had no effect on the TGF-β-induced levels of TIMP-3 mRNA. In addition, adenoviral delivery of Smad3DN potently inhibited the up-regulatory effect of TGF-β on TIMP-3 expression (Figure 3A).

To further dissect the role of Smad signaling in TGF-β-elicited TIMP-3 gene expression, we analyzed TIMP-3 expression also on the protein level with immunoblotting. In accordance with the results above, a 24-h TGF-β stimulation of empty control virus (RAdpCA3) infected fibroblasts resulted in marked induction of TIMP-3 production, as compared to untreated control cells (Figure 3B). In addition, overexpression of Smad3 enhanced the up-regulatory effect of TGF-β. In contrast, adenoviral expression of the inhibitory Smad, Smad7, and Smad3DN potently suppressed the TGF-β-elicited induction of TIMP-3 production (Figure 3B). Together, these observations provide evidence, that Smad signaling in particular *via* Smad3 mediates the TGF-β-elicited induction of TIMP-3 gene expression in human fibroblasts.

### Smad3, p38, and ERK1/2 Cooperate in Regulating TIMP-3 Expression

**Figure 4:**
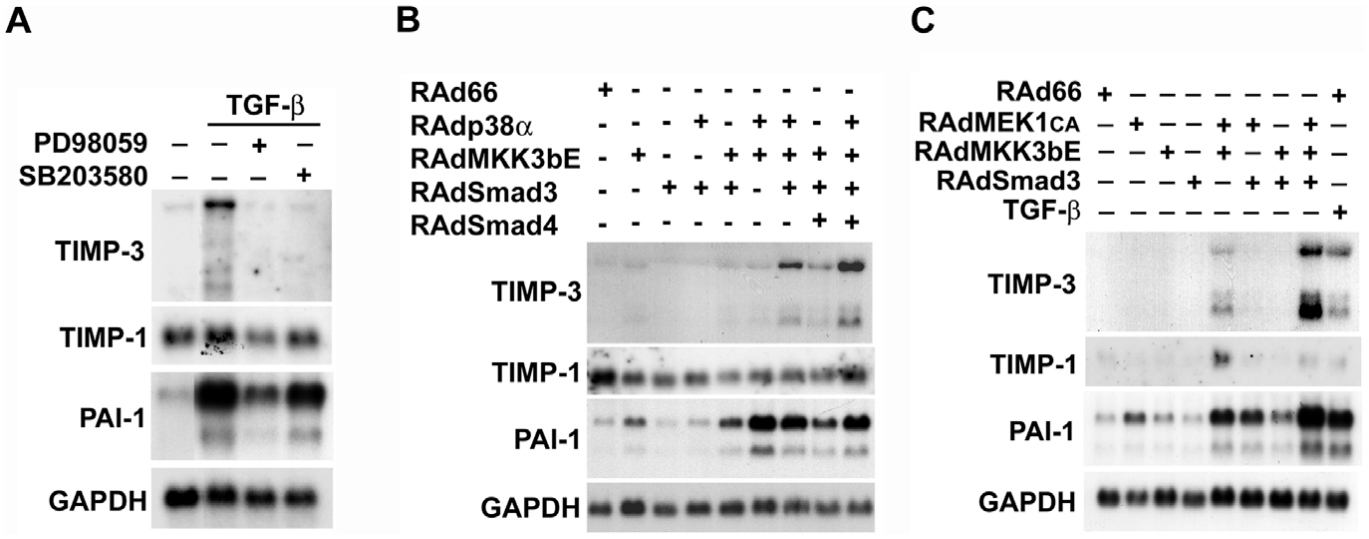
Smad3, p38α and ERK1/2 cooperate in the induction of TIMP-3 gene expression in human fibroblasts. (**A**) Human gingival fibroblasts were serum starved for 18 h, and treated for 1 h with PD98059 (30 µM), or SB203580 (10 µM), specific chemical inhibitors for MEK1 or p38, respectively. Subsequently, TGF-β1 (5 ng/ml) was added, and the cultures incubated for 16 h. Total cellular RNAs were harvested and analyzed for the levels of TIMP-3, TIMP-1, PAI-1 and GAPDH mRNAs by Northern blot hybridizations. (**B**) Human gingival fibroblasts were transduced with recombinant adenoviruses for wild-type p38α (RAdp38α), constitutively active MKK3b (RAdMKK3bE), Smad3 (RAdSmad3), Smad4 (RAdSmad4), or with empty control virus (RAd66) at MOI 500, and incubated for 24 h. Total cellular RNA was analyzed with Northern blot hybridizations for the expression of TIMP-3, TIMP-1, PAI-1, and GAPDH mRNAs. (**C**) Human gingival fibroblasts were transduced with recombinant adenoviruses for constitutively active MEK1 (RAdMEK1CA), constitutively active MKK3b (RAdMKK3bE), Smad3 (RAdSmad3) and control virus RAd66 as in (**B**). Total cellular RNA was analyzed with Northern blot hybridizations for the expression of TIMP-3, TIMP-1, PAI-1, and GAPDH mRNAs.

Smad signaling is regulated through crosstalk with other signaling cascades, *e.g.* MAPK pathways p38, ERK1/2, and JNK, and Cam kinase II [20]. TGF-β activates ERK1/2 and p38 MAPK pathways in gingival fibroblasts [21]. In addition, our previous observations demonstrate, that Smad3 co-operates with p38 MAPK in regulating the expression of MMP-13 [25], and with ERK1/2 in the TGF-β-elicited expression of connective tissue growth factor (CTGF) in human gingival fibroblasts [26]. Therefore, we first examined whether ERK1/2 and p38 MAPK pathways play a role in mediating the effect of TGF-β on TIMP-3 gene expression in human gingival fibroblasts. We used PD98059 (30 µM), an inhibitor for MEK1, the upstream activator of ERK1/2, and SB203580 (10 µM), a specific chemical inhibitor for p38 MAPK. Interestingly, PD98059 and SB203580 potently down-regulated TGF-β-induced TIMP-3 mRNA expression (Figure 4A), indicating that both p38 and ERK1/2 MAPKs are crucial for mediating the effects of TGF-β on TIMP-3 expression. Treatment with PD98059 suppressed also TGF-β-induced PAI-1 expression, whereas SB20358 had a weaker inhibitory effect. In comparison, PD98059 and SB203580 had modest effects on the TGF-β-induced levels of TIMP-1 (Figure 4A).

Next, we examined the possible crosstalk between the MAPK and Smad3 pathways in the regulation TIMP-3 gene expression. Human gingival fibroblasts were first infected with recombinant adenoviruses for Smad3 and Smad4 together with adenoviruses for wild-type p38α (RAdp38α), and constitutively active MKK3b (RAdMKK3bE), an upstream activator of p38, and incubated for 24 h. As shown in Figure 4B, the activation of p38α by MKK3bE and simultaneous co-expression of Smad3 resulted in the induction of TIMP-3 mRNA expression in the absence of TGF-β. This effect was further augmented by simultaneous co-expression of Smad4 (Figure 4B). This indicates, that activation of p38 and simultaneous co-expression of Smad3 and Smad4 can induce the expression of endogenous TIMP-3 even in the absence of TGF-β. Activation of p38α by MKK3bE resulted in induction of PAI-1 mRNA, but this was not further augmented by co-expression Smad3 and Smad4 (Figure 4B). This indicates, that p38α participates in regulating the expression of PAI-1, but does not co-operate with Smad3 in this respect. On the contrary, the levels of TIMP-1 mRNA were not markedly altered under these conditions.

Next, gingival fibroblasts were transduced with adenoviruses for constitutively active MEK1 (RAdMEK1CA) alone or in combination with RAdSmad3 and RAdMKK3bE. Activation of endogenous p38 by RAdMKK3bE or ERK1/2 by RAdMEK1CA alone was not sufficient to induce TIMP-3 expression, but combined activation of both ERK1/2 and p38 resulted in potent induction in the expression of TIMP-3 mRNA (Figure 4C). Furthermore, overexpression of Smad3 augmented this effect. The basal level of TIMP-1 mRNA was relatively low under these conditions, and only activation of both ERK1/2 and p38 by RAdMEK1CA and RAdMKK3bE, respectively, induced the expression of TIMP-1 mRNA (Figure 4C). In the same cells, the level of PAI-1 mRNA was upregulated when ERK1/2 was activated by MEK1CA, and this was further augmented by overexpression of Smad3 and by activation of p38 by MKK3bE (Figure 4C), suggesting that p38, ERK1/2 and Smad3 co-operate in mediating the induction of PAI-1 expression.

## Discussion

TGF-β plays an important role in regulation of ECM homeostasis. It controls both the deposition and turnover of ECM components, such as the fibrillar collagens and fibronectin, and inhibits the expression of matrix degrading proteolytic enzymes, such as serine proteinases and MMPs. In addition, TGF-β induces the production of proteinase inhibitors, including PAI-1 and TIMPs [12], [13], [16]. As specific inhibitors of metalloproteinases, TIMPs are essential for maintaining the balance between ECM deposition and degradation in both physiological and pathological conditions. Smads are crucial in mediating many cellular actions of the TGF-β family including ECM gene expression, *e.g.* type I and VII collagen, aggrecan, PAI-1, and MMP-13 [25], [43], [45]–[47]. Furthermore, Smads participate in the down-regulation of human *MMP1* promoter activity by TGF-β [19].

In this study, we have elucidated the cellular signaling pathways involved in mediating the TGF-β-induced expression of TIMP-3, an ECM component, by fibroblasts. Our observations demonstrate that TIMP-3 is clearly a TGF-β responsive gene. Its expression by fibroblasts was found to be up-regulated by TGF-β more potently than the expression of TIMP-1. Furthermore, we observed that the expression of TIMP-3 was dependent on Smad signaling. This was confirmed by using Smad4-deficient murine fibroblasts where TIMP-3 mRNA expression was completely abolished as compared to corresponding wild-type fibroblasts and rescued by restoration of Smad4 expression. Overexpression of Smad3 in human gingival fibroblasts resulted in enhanced expression of TIMP-3 in response to TGF-β, whereas dominant negative Smad3 and Smad7 suppressed TIMP-3 expression, providing evidence that Smad3 specifically mediates the TGF-β-elicited induction of TIMP-3 expression. This is in accordance with previous observations showing that Smads mediate TGF-β-stimulated TIMP-3 expression in human chondrocytes and that TIMP-3 gene is a target of Smad signaling pathway [14].

We have previously observed that Smad3 mediates the TGF-β-induced expression of MMP-13 and CTGF in human gingival fibroblasts and squamous carcinoma cells [24]–[26]. In addition, there are other reports demonstrating that Smad3 is crucial in mediating the effects of TGF-β on ECM deposition and turnover [16], [20]. TIMP-3 gene expression is also upregulated in human scleroderma fibroblasts, and it is further enhanced by TGF-β, suggesting that TIMP-3 as an ECM component is involved in the pathogenesis of dermal fibrosis [15]. Given the documented role of Smad signaling in tissue fibrosis [48], it is conceivable, that the Smad3-mediated up-regulation of TIMP-3 expression may play a role in excessive accumulation of ECM and subsequent development of tissue fibrosis. In addition, recent obervations implicate TIMP-3 in the regulation of inflammation following tissue injury, suggesting an important role for TIMP-3 in the process of normal tissue repair [49]. Furthermore, stromal TIMP-3 has recently been show to regulate basal lymphocyte populations in liver tissue and prevent autoimmune hepatitis providing further evidence for the role of TIMP-3 in regulation of inflammation [50]. The results of the present study suggest a novel indirect anti-inflammatory mechanism for TGF-β by inducing TIMP-3 expression by fibroblasts in injured tissue. It is likely, that the signaling mechanisms documented here also play a crucial role in regulating this anti-inflammatory function of TIMP-3 in tissue repair.

There is a considerable body of evidence concerning the crosstalk between the distinct cell signaling cascades activated by TGF-β, *e.g.* MAPK and Smad pathways. ERK1/2, JNK, and p38 MAPKs can activate or inhibit the Smad signaling pathway by phosphorylating Smad2 or Smad3 [22], [23], [51], [52]. In addition, delayed phosphorylation of p38 MAPK by TGF-β has been shown to be mediated by the Smad pathway [27]–[29]. Recent studies have shown, that coordinate activation of Smad and MAPK pathways plays an important role in epithelial-mesenchymal transition and myofibroblast formation induced by TGF-β [53], [54]. Furthermore, Smad3 is inactivated via hypoxia-induced dephosphorylation by protein phosphatase 2A in epithelial cells [55].

**Figure 5:**
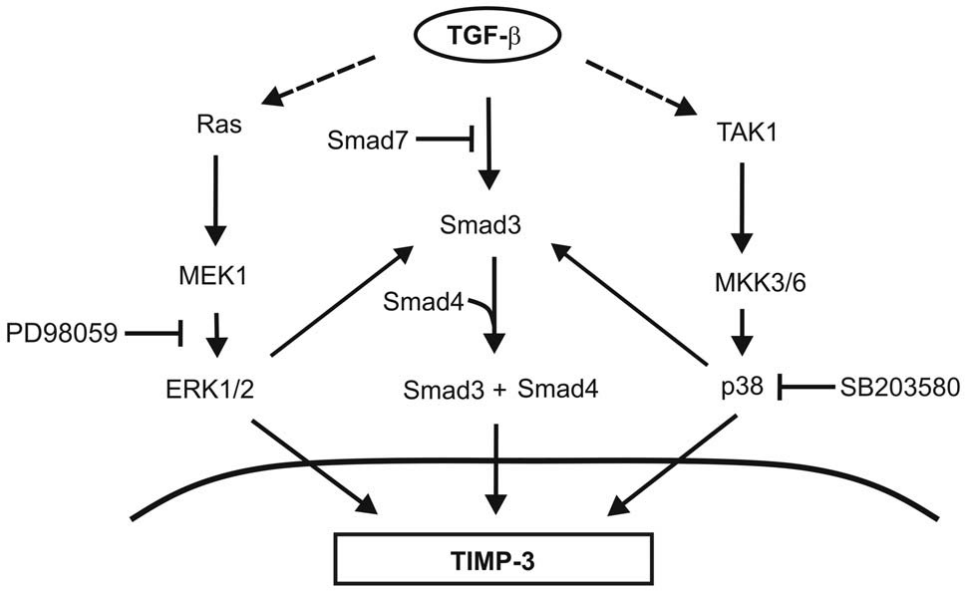
A schematic representation of the complex interplay of TGF-β-signaling pathways regulating TIMP-3 expression in human fibroblasts. Stimulation of human gingival fibroblasts with TGF-β results in activation of Smad3, ERK1/2 and p38 MAPK pathways. Activation of all three pathways is required for induction of TIMP-3 expression by TGF-β Smad3 associates with Smad4 and mediates induction of TIMP-3 expression by TGF-β. ERK1/2 and p38 MAPK pathways both co-operate with Smad3 in mediating the induction of TIMP-3 expression by TGF-β.

Here, p38 and ERK1/2 MAPK pathways mediated the effects of TGF-β on TIMP-3 gene expression in human gingival fibroblasts, since blocking their activity with chemical inhibitors SB203580 and PD98059 resulted in a marked suppression of TGF-β-induced TIMP-3 mRNA levels. In addition, activation of p38 by MKK3bE and ERK1/2 by MEK1CA in combination resulted in the induction of TIMP-3 expression in the absence of TGF-β, and this effect was augmented by simultaneous co-expression of Smad3. This indicates that p38, ERK1/2, and Smad3 synergistically mediate the up-regulation of the expression of TIMP-3. We have previously demonstrated, that p38 and Smad3 co-operate in mediating TGF-β-induced expression of MMP-13 in human gingival fibroblasts [25]. Activated p38 induced activation and nuclear translocation of Smad3 in gingival fibroblasts, indicating that p38 MAPK is able to activate Smad3. In addition, ERK1/2 and Smad3 co-operatively mediated the TGF-β-induced CTGF gene expression [26]. It is conceivable that also here, p38α and ERK1/2 influenced Smad3 activation, and together these signaling mediators induced TIMP-3 gene expression. The results are summarized Figure 5 demonstrating the complex regulation of TIMP-3 gene expression by crosstalk between ERK1/2, p38, and Smad3 pathways.

To conclude, these results demonstrate that coordinate activation of Smad3, p38α, and ERK1/2 is essential for the induction of TIMP-3 expression by TGF-β. In addition, this study demonstrates that complex crosstalk between Smad3 and ERK1/2 and p38 MAPK pathways plays a pivotal role in mediating the signals triggered by TGF-β in fibroblasts, and in controlling ECM deposition in *e.g.* tissue repair and fibrosis.

## Supporting Information

Table S1 **Primer and probe sequences for Quantitative Reverse Transcription PCR of mouse TIMP-3 and PAI-1 mRNAs.**
(DOCX)
