# Supplementary material for: TGF-β-Elicited Induction of Tissue Inhibitor of Metalloproteinases (TIMP)-3 Expression in Fibroblasts Involves Complex Interplay between Smad3, p38α, and ERK1/2
Source: PLoS One. 2013 Feb 28;8(2):e57474. doi: 10.1371/journal.pone.0057474 (PMC3585359; doi:10.1371/journal.pone.0057474)
Supplement: Table S1 — Primer and probe sequences for Quantitative Reverse Transcription PCR of mouse TIMP-3 and PAI-1 mRNAs. (DOCX) [file pone.0057474.s001.docx]

**Table S1. Primer and probe sequences for Quantitative Reverse Transcription PCR of**

**mouse TIMP-3 and PAI-1 mRNAs**

| **Gene** | **Primer/Probe** | **Sequence** |
| --- | --- | --- |
| TIMP-3 | Forward Primer:  Reverse Primer:  Probe: | 5'-GGCCTCAATTACCGCTACCA-3'  5'-CTGATAGCCAGGGTACCCAAAA-3'  5'-FAM-TGCTACTACTTGCCTTGTTTTGTGACCTCCA-TAMRA-3' |
| PAI-1 | Forward Primer:  Reverse Primer:  Probe: | 5’-CCGTCTCTGTGCCCATGAT -3’  5’-GGCAGTTCCACGACGTCATA -3’  5’-FAM-CTCAGAGCAACAAGTTCAACTACACTGAGTTCACC -3’-TAMRA |
